# Primary thyroid tuberculosis mimicking papillary carcinoma of thyroid: a rare case report

**DOI:** 10.1097/MS9.0000000000000648

**Published:** 2023-04-11

**Authors:** Sabin Banmala, Sarita K.C., Manita Raut, Rajesh Poudel, Sabin Karki, Suman Maharjan

**Affiliations:** a Nepalese Army Institute of Health Sciences – College of Medicine; b Department of Otorhinolaryngology, Shree Birendra Hospital, Kathmandu, Nepal

**Keywords:** case report, *Mycobacterium tuberculosis*, thyroid malignancy, thyroid tuberculosis, tubercular thyroiditis

## Abstract

Primary thyroid tuberculosis is an extremely rare extrapulmonary involvement by the causative agent, *Mycobacterium tuberculosis*. Its rarity and resemblance to thyroid malignancy led to unnecessary aggressive operative interventions.

**Case Presentation:**

A 54-year-old female presented with recent onset dysphagia and foreign body sensation in the throat for 3 months, and anterior neck swelling since last 10 years.

**Clinical Findings and Investigations:**

A single nodular firm anterior neck swelling was present which moves with deglutition. Thyroid function test was normal. Ultrasonography thyroid revealed TIRADS-3. Fine-needle aspiration cytology was suggestive of papillary carcinoma of thyroid.

**Interventions and Outcome:**

Total thyroidectomy with central compartment neck dissection was performed. Histopathology of the thyroid specimen revealed tubercular thyroiditis. Postoperatively, Mantoux test and interferon gamma radioassay were positive. Antitubercular therapy was given for total of 6 months.

**Conclusions:**

With ultrasonography-guided fine-needle aspiration cytology, preoperative diagnosis of primary thyroid tuberculosis is quite challenging even in tuberculosis endemic countries. So, it should be considered one of the differential diagnoses despite negative relevant history and without clinical cervical lymph nodes involvement with cytology proven suspicious papillary thyroid cancer before proceeding for surgical intervention.

## Introduction

Highlights

- Even in tuberculosis (TB) endemic countries, primary thyroid tuberculosis (TTB) is an extremely rare condition.
- Primary involvement of thyroid gland by *Mycobacterium tuberculosis* bacilli is much more uncommon and even more difficult to diagnose.
- Surgery is sometimes inevitable as primary TTB being extremely rare, mimics thyroid malignancy and fine-needle aspiration cytology (FNAC) may be inadequate for the diagnosis.
- A preoperative diagnosis of the disease is quite difficult, but if detected can help avoid surgery in many patients.

TB remains a worldwide public health problem with an estimated 10 million people infected with TB and a total of one and half million death from TB^1^. The causative agent of TB, *M. tuberculosis* mainly causes pulmonary TB, however, extrapulmonary TB has increased in the past few decades which is estimated to be 20% of all TB cases^2^–^4^. Extrapulmonary TB has been described in various parts of the body. However, even in TB endemic countries, primary TTB with histopathological and microbiological evidence and without evidence of extra-TTB is an extremely rare condition^3^,^5^. TB in such atypical locations is usually misdiagnosed resulting in diagnostic dilemma and unnecessary aggressive therapeutic interventions^6^.

We here present a rare case of primary TTB mimicking papillary carcinoma of thyroid which was diagnosed by histopathology and microscopy after total thyroidectomy.

This case has been reported in line with the SCARE 2020 criteria^7^.

## Case presentation

**Figure 1:**
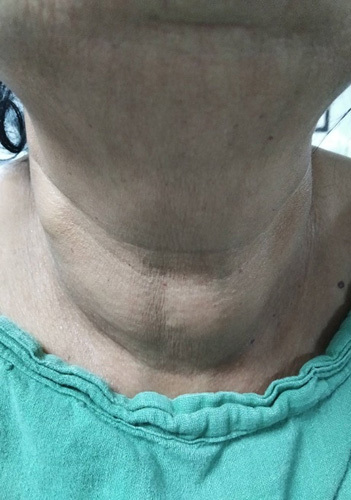
Female with thyroid swelling.

A 54-year-old female presented to Ear, Nose, and Throat Outpatient Department with complaints of left-sided anterior neck swelling for the past 10 years and recently developed dysphagia and foreign body sensation in throat for the past 3 months. There was no history of fever, significant weight loss, night sweats, change in bowel habits, sleep pattern disturbances, palpitations. She is a known case of hypertension under amlodipine tablets 5 mg once a day over last 5 years. There was no past history of TB, thyroid diseases, and no family history of TB and thyroid disorders. On physical examination, a single nodular, round-shaped nontender swelling of size 2×3.5 cm with regular margin, firm consistency was detected on the left anterior neck which moves with deglutition (Fig. 1). The overlying skin appeared normal with no signs of inflammation. Lymph nodes were not palpable. Her vital parameters and systemic examination including chest, cardiovascular, and per abdominal examinations were normal.

Thyroid function test was normal. Ultrasonography (USG) of the neck revealed a single solid cystic lesion (16×20 mm) in left lobe with evidence of echogenic contents within cystic portion, however, no vascularity was noted (TIRADS-3). Bilateral thyroid lobe showed few variable sized isoechoic to hypoechoic solid lesions without peripheral or central vascularity.

USG-guided FNAC of the left thyroid lobe was performed which showed atypical thyroid follicular cells in monolayer sheet and papillary arrangements with scanty and viscous colloid. Each atypical cell showed nuclear crowding, overlapping, nuclear grooving and Hurthle cell changes. These findings increased suspiciousness of papillary carcinoma of thyroid, Bethesda category V. Since our institution does not have the protocol to do the core biopsy for thyroid lesion due to lack of expertise, we did USG-guided FNAC.

**Figure 2:**
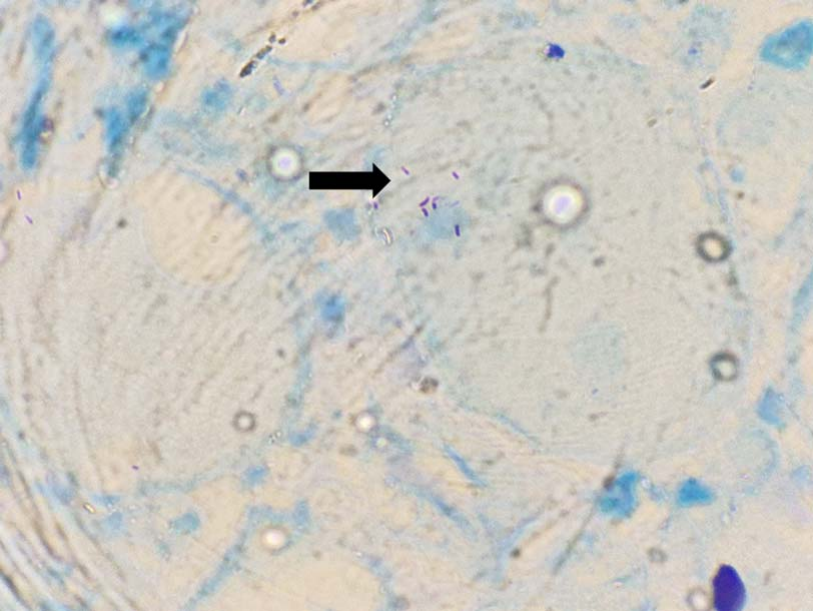
AFB seen in surgically removed thyroid specimen (black arrow).

**Figure 3:**
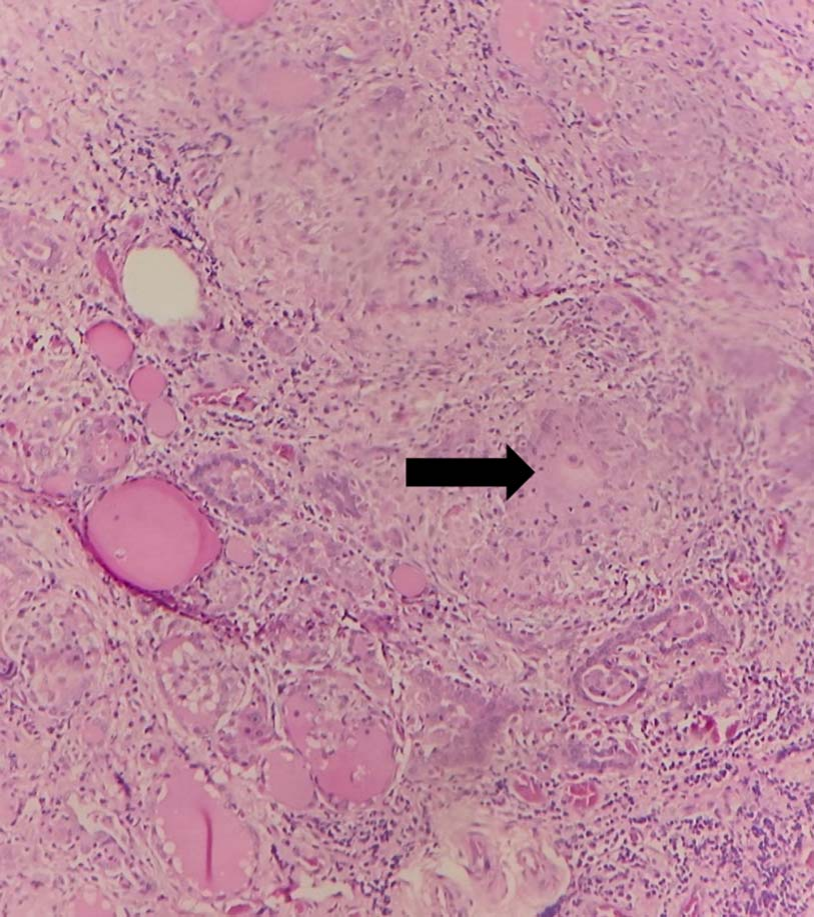
Histopathology examination of tubercular thyroiditis showing necrosis (black arrow).

Taking into account the sudden onset symptoms of dysphagia and foreign body sensation in the throat, clinical finding of firm nodular thyroid swelling, US finding of solid cystic lesion, and FNAC findings suggestive of papillary carcinoma of thyroid, surgical treatment was undertaken. Total thyroidectomy with central compartment neck dissection was performed. Intraoperatively, enlarged thyroid gland (right lobe>left lobe) with multiple nodules on right lobe largest measuring 2×2 cm and on left lobe largest measuring 1.5×1 cm were noted. The final diagnosis was established through definitive histopathological examination. Histological evaluation revealed well-formed granuloma comprising of multinucleated giant cells, lymphocytes and epitheloid histiocytes in the background of caseous necrosis. Zeihl–Neelsen stain showed that specimens from both thyroid tissue and lymph nodes were positive for acid-fast bacilli (Figs. 2 and 3). The final diagnosis made was primary tubercular thyroiditis.

There were no postoperative complications. l-thyroxine, calcium, and vitamin D supplements were given. Mantoux test, sputum test, high-resolution computed tomography chest and interferon gamma radioassay were done. Mantoux test showed 20 mm induration which showed strong positive and interferon gamma radioassay was also positive, however, sputum test and high-resolution computed tomography chest were normal. Thus, patient was treated with antitubercular therapy (2 months of isoniazid, rifampicin, pyrazinamide, and ethambutol, and 4 months of isoniazid and rifampicin). At present, she has completely recovered.

## Discussion

TTB is rare even in countries where TB is common, and primary disease is even rarer with estimated frequency of 0.1–0.4% of all TB cases^8^,^9^. TTB mostly occur either through the hematogenous spread from elsewhere in the body or direct spread from adjacent foci or the lymphatic route. However, primary involvement of thyroid gland by *M. tuberculosis* bacilli are much more uncommon and even more difficult to diagnose^6^,^9^. The rarity of TTB has been attributed to the bactericidal action of the thyroid colloid, the extensive vascularization leading to high oxygenation and high levels of iodine in the thyroid gland^10^. TTB has slight female predominance with mean age at onset being around third to fourth decade^9^.

The pathology of TTB may be as follows: (1) multiple lesions throughout the gland-like miliary TB; (2) enlargement of gland due to caseating granulomas; (3) cold abscess formation sometimes with multiple sinuses; (4) chronic fibrosing TB, difficult to distinguish from De Quervein’s thyroiditis; (5) acute abscess formation, when there is a danger of making wrong diagnosis of carcinoma^11^.

Clinically, patients of TTB complain of variable symptoms ranging from thyroid nodules, diffuse or multinodular goiter, cold or acute abscess, pressure symptoms like dyspnea, hoarseness, and cervical lymphadenopathy. Constitutional symptoms of pulmonary TB like fever, fatigue, night sweats, and weight loss are almost never seen in cases of TTB^5^. Patients with TTB usually have normal level of thyroid hormones (T3, T4, thyroid-stimulating hormone). However, there have been few cases of thyrotoxicosis or myxedema reported due to thyroid destruction by TB^5^,^8^.

The imaging techniques are not very helpful because of nonspecific findings of disease and also because of the rare occurrence^2^,^9^. Thyroid ultrasound may reveal appearances ranging from multifocal, ill-defined, heterogeneous hypoechoic lesions involving both lobes of the thyroid, to solitary well-defined, heterogeneous, predominantly anechoic lesion with internal echoes and irregular margins/walls^12^. Contrary to above mentioned, single solid cystic lesion (16×20 mm) in left lobe with evidence of echogenic contents within and few variable sized isoechoic to hypoechoic solid lesions in bilateral thyroid lobes were present in our case. Computed tomography and MRI are also nonspecific to differentiate malignancy from TTB^13^.

USG-guided FNAC is important and inexpensive method of diagnosis that helps in avoiding unnecessary surgery^2^,^14^. Fine-needle aspirates from tubercular thyroid shows epithelioid granulomas with necrosis. However, the demonstration of acid-fast bacilli and the presence of caseating necrosis are pathognomonic for tuberculous thyroiditis as epitheloid granuloma may be seen in other conditions like granulomatous thyroiditis, fungal infection, sarcoidosis, vasculitis, foreign body reactions, plasma cell neoplasms, and even in anaplastic carcinoma^13^,^15^.

Surgery is sometimes inevitable as primary TTB is extremely rare, mimics thyroid malignancy and FNAC may be inadequate for diagnosis. Thus, majority of cases are diagnosed postoperatively by histopathological examination of the surgical specimen as in our patient. On histopathological examination, TTB reveals epitheliod cell granulomas with central caseous necrosis, peripheral lymphocytic infiltration and Langerhan’s giant cells^16^.

We performed surgery in this case as the physical examination findings, imaging and FNAC were highly suspicious in favor of malignancy. The surgery is not a completely unnecessary step for our patient as TTB may coexist with thyroid carcinoma and without surgery the diagnosis could have been missed^3^,^17^.

TTB is primarily treated by antitubercular medications like any other extrapulmonary TB and where the response to antitubercular therapy is satisfactory. However, a thyroidectomy should be performed on large masses causing pressure symptoms, TTB cases coexisting with thyroid carcinoma or suspicious malignant lesions^2^,^5^,^9^,^14^ /WHO recommendation for TTB is 2 months of isoniazid, rifampicin, pyrazinamide, and ethambutol followed by 4 months of isoniazid and rifampicin^18^.

## Conclusions

TTB is an extremely rare form of extrapulmonary TB and quite challenging to diagnose preoperatively. Even with USG-guided FNAC, preoperative diagnosis of primary TTB is quite challenging even in TB endemic countries. So, it should be considered one of the differential diagnoses of thyroid nodule that resembles malignancy despite negative relevant history and without clinical cervical lymph nodes involvement with cytology proven suspicious papillary thyroid cancer before proceeding for surgical intervention. Performing other tests like PCR from fine-needle aspiration fluid in TB endemic countries’ hospitals which do not have high volume thyroid cases should be considered to avoid unnecessary surgeries.

## Ethical approval

Not applicable.

## Consent

Written informed consent was obtained from the patient for publication of this case report and accompanying images. A copy is available for review by the Editor in chief of this journal on request.

## Sources of funding

No funding was required for the publication of this case report.

## Conflicts of interest disclosure

The authors declare that they have no financial conflict of interest with regard to the content of this report.

## Research registration unique identifying number (UIN)

Not applicable.

## Provenance and peer review

Not commissioned, externally peer reviewed.
